# PA21, a novel phosphate binder, improves renal osteodystrophy in rats with chronic renal failure

**DOI:** 10.1371/journal.pone.0180430

**Published:** 2017-07-13

**Authors:** Atsushi Yaguchi, Satoshi Tatemichi, Hiroo Takeda, Mamoru Kobayashi

**Affiliations:** Pharmacology Research Laboratory, R&D., Kissei Pharmaceutical Co., Ltd., Azumino-City, Nagano-Pref., Japan; University of Milan, ITALY

## Abstract

The effects of PA21, a novel iron-based and non-calcium-based phosphate binder, on hyperphosphatemia and its accompanying bone abnormality in chronic kidney disease-mineral and bone disorder (CKD-MBD) were evaluated. Rats with adenine-induced chronic renal failure (CRF) were prepared by feeding them an adenine-containing diet for four weeks. They were also freely fed a diet that contained PA21 (0.5, 1.5, and 5%), sevelamer hydrochloride (0.6 and 2%) or lanthanum carbonate hydrate (0.6 and 2%) for four weeks. Blood biochemical parameters were measured and bone histomorphometry was performed for femurs, which were isolated after drug treatment. Serum phosphorus and parathyroid hormone (PTH) levels were higher in the CRF rats. Administration of phosphate binders for four weeks decreased serum phosphorus and PTH levels in a dose-dependent manner and there were significant decreases in the AUC_0–28 day_ of these parameters in 5% PA21, 2% sevelamer hydrochloride, and 2% lanthanum carbonate hydrate groups compared with that in the CRF control group. Moreover, osteoid volume improved significantly in 5% of the PA21 group, and fibrosis volume and cortical porosity were ameliorated in 5% PA21, 2% sevelamer hydrochloride, and 2% lanthanum carbonate hydrate groups. These results suggest that PA21 is effective against hyperphosphatemia, secondary hyperparathyroidism, and bone abnormalities in CKD-MBD as sevelamer hydrochloride and lanthanum carbonate hydrate are, and that PA21 is a new potential alternative to phosphate binders.

## Introduction

A decline in kidney function due to chronic kidney disease (CKD) causes mineral disorders. It leads to systemic abnormalities such as vascular calcification and affects prognosis. All pathologies associated with CKD were unified and the concept of CKD-mineral and bone disorder (CKD-MBD) was suggested [1].

Metabolism disorders of phosphorus are responsible for the onset of CKD-MBD, because reduced renal function and the accompanying phosphorus accumulation promote the secretion of fibroblast growth factor 23 (FGF23) and parathyroid hormone (PTH) and the inhibition of 1α,25-dihydroxy-vitamin D_3_ (1,25(OH)_2_D_3_) synthesis. In addition, they also result in the development of secondary hyperparathyroidism (SHPT), ectopic calcification, and bone lesion through PTH hypersecretion and mineral metabolism disorders [2, 3]. Furthermore, hyperphosphatemia, which is a cause of CKD-MBD, also contributes to cardiovascular risk and mortality [4]. Therefore, it is thought that the control of serum phosphorus levels is extremely important in the treatment of CKD-MBD.

Besides hyperphosphatemia, the development of ectopic calcification and bone disease also increases the risk of death, because vascular calcification was a significant predictor of cardiovascular death [5, 6], and the fracture that bone disease may lead to was associated with mortality [7, 8]. Thus, these events need to be controlled adequately. In the diagnosis of ectopic calcification and bone disease in CKD patients, the evaluation of aorta and femoral artery calcification can be conducted noninvasively with an X-ray and CT scan while the method to correctly diagnose bone lesion histopathologically has to rely on bone biopsy, which is an invasive method. However, repetition of the bone biopsy is not realistic and is thus not recommended in daily practice [9]. Subsequently, it is useful to evaluate these effects on bone lesion histopathologically with diseased animal models reflecting clinical pathology in order to presume the effects on renal osteodystrophy (ROD) in CKD patients.

To improve the prognosis of a patient with CKD-MBD, adequate control of serum phosphorus levels is important [10]. Phosphorus removal with dialysis and the dietary restriction of phosphorus intake can be considered the best phosphorus-management option in patients with end-stage renal disease, who lose phosphorus excretion function; however, these options are insufficient in themselves to control serum phosphorus levels adequately [11]. Moreover, dietary restriction can deteriorate patients’ nutritional status and increase mortality conversely [12]. Thus, many patients need phosphate binders for control of phosphorus levels.

PA21 (sucroferric oxyhydroxide) is a new, iron-based, non-calcium-based phosphate binder and consists of iron (III) oxyhydroxide, sucrose, and starches. PA21, as well as other phosphate binders, exhibits reducing effects on serum phosphorus levels by combining with phosphates derived from food and inhibiting phosphate absorption in the gastrointestinal (GI) tract [13]. Recent clinical studies indicated that it reduced serum phosphorus levels remarkably [14]. Additionally, it is reported that PA21 decreased serum phosphorus and PTH levels in addition to suppressing vascular calcification in a non-clinical study with CRF rats, while it did not repress increased serum creatinine level, which characterizes the development of CRF [15, 16]. However, studies detailing this effects in ROD are not reported.

Therefore, in this study, we investigated the effect of PA21 on ROD using adenine-induced CRF rats and compared the effect of PA21 with that of sevelamer hydrochloride and lanthanum carbonate hydrate.

## Materials and methods

### Drugs used

PA21 was provided by Vifor Pharma (Glattbrugg, Switzerland). Sevelamer hydrochloride and lanthanum carbonate hydrate were purchased from AK Scientific, Inc. (Union city, CA, USA) and Alfa Aesar (Ward hill, MA, USA), respectively.

### Experimental animals

Eight-week-old male Sprague-Dawley (SD) rats were purchased from Charles River Laboratories Japan, Inc. (Yokohama, Japan). Rats were maintained individually in suspended metal cages and fed a CE-2 diet (1.09% (1.08–1.10%) calcium, 1.06% (1.04–1.07%) phosphorus, 25.6% (25.4–25.8%) crude protein, and 2.1 IU/g vitamin D_3_) (CLEA Japan, Inc., Tokyo, Japan) during an acclimatization period. All animals were observed for clinical signs at least once a day. This study was performed in accordance with the guidelines approved by the Laboratory Animal Committee of Kissei Pharmaceutical Co., Ltd., which conform to current Japanese law.

### Study design

The study design is shown in Table 1. After an acclimatization period, the normal group of 12 rats continued to be fed with a normal CE-2 diet throughout the study. As reported previously [17], the disease group, comprising of 128 rats, were fed with a CE-2 diet containing 0.75% adenine (0.75% adenine diet, CLEA Japan, Inc.) for 14 days. Then, this group was fed with a CE-2 diet containing 0.5% adenine (0.5% adenine diet, CLEA Japan, Inc.) for the next 14 days.

**Table 1:** Study design.

Day	1	14		28	56
Group	Disease model period				Treatment period
Normal	CE-2				CE-2
Control	0.75% adenine (diet)		0.5% adenine (diet)		CE-2
0.5% PA21					0.5% PA21 (diet)
1.5% PA21					1.5% PA21 (diet)
5% PA21					5% PA21 (diet)
0.6% SH					0.6% sevelamer hydrochloride (diet)
2% SH					2% sevelamer hydrochloride (diet)
0.6% LC					0.6% lanthanum carbonate hydrate (diet)
2% LC					2% lanthanum carbonate hydrate (diet)

0.75% adenine diet was administered to the eight groups from day 1 to day 14, then 0.5% adenine diet was administered from day 15 to day 28. At day 29, administration of the investigated drugs was started. PA21 was administered at 0.5%, 1.5% and 5% mixed diet for four weeks. Sevelamer hydrochloride and lanthanum carbonate hydrate were administered 0.6% and 2% mixed diet for four weeks.

After the disease model period, based on the results of blood biochemistry (serum phosphorus levels and serum urea-nitrogen (SUN) levels) and body weights at day 28, 10 rats were selected from the normal group. From the adenine-treated group, as well, based on the results of these parameters at day 28, 80 rats were selected and divided into eight groups. After the grouping, rats in each group were fed with a CE-2 diet (control group) or with a CE-2 diet containing either PA21 (0.5, 1.5, and 5%), sevelamer hydrochloride (0.6 and 2%), or lanthanum carbonate hydrate (0.6 and 2%) for 28 days (as shown in Table 1).

Body weight and food intake volume were measured weekly, and blood samples were collected on days 0, 7, 14, 28, 31, 35, 42, 49, and 56 in order to measure the serum parameters over time. On day 57, all surviving rats were sacrificed by abdominal aortic puncture under anesthesia.

### Blood biochemistry

SUN, serum creatinine, phosphorus, and calcium levels were determined with an automatic clinical chemistry analyzer (JCA-BM6010, JEOL Ltd., Tokyo, Japan). Serum PTH levels were determined by using the rat PTH IRMA kit (Immutopics, Inc., San Clemente, CA, USA).

### Bone histomorphometry

At the end of the study, the femurs were isolated and fixed in 10% neutral-buffered formalin. The femurs were embedded in a methyl methacrylate resin without decalcification and sectioned on the frontal plane. Cross-sections, 6 μm thick, were obtained by the Wet method with a fully automatic rotary microtome (RM2255, Leica Microsystems, WA, USA). The sections were de-resinated with xylene and stained with a Villanueva-Goldner stain.

Bone histomorphometry was performed with a bone histomorphometry system (Histometry RT CAMERA Ver. 1.33, System supply Co., Ltd., Nagano, Japan) and a System biological microscope (BX53, Olympus Corporation, Tokyo, Japan). Histomorphometry of the trabecular bone was performed in a 1.71 mm × 2.28 mm area. The region of interest was determined by locating to around 0.8 mm proximal from the growth plate cartilage. The histomorphometric parameters of the trabecular bone were measured by using a × 20 objective. The cortical bone area starting at a distance of 2 mm from the growth plate cartilage and extending by 1.725 mm proximally was subjected to measurement. The histomorphometric parameters of the cortical bone were measured by using a × 10 objective. Primary parameters were measured and secondary parameters were calculated with the primary parameters. The measured variables are shown in Table 2. Sample images were obtained with a BZ-X700 microscope (KEYENCE Corporation, Osaka, Japan).

**Table 2:** Bone histomorphometric parameters measured in this study.

Parameter (unit)	Explanation
**Primary parameters of trabecular bone**	
BS (mm)	Trabecular bone surface
BV (mm^2^)	Trabecular bone volume
OV (mm^2^)	Osteoid volume
Ob.S (mm)	Osteoblast surface
Oc.S (mm)	Osteoclast surface
Fb.V (mm^2^)	Fibrosis volume
**Secondary parameters of trabecular bone**	
OV/BV (%)	Percentage of trabecular bone volume consisting of osteoid
Ob.S/BS (%)	Percentage of trabecular bone surface covered by osteoblast
Oc.S/BS (%)	Percentage of trabecular bone surface covered by osteoclast
**Primary parameters of cortical bone**	
Ct.BV (mm^2^)	Cortical bone volume
Vo.Ar (mm^2^)	Void area
**Secondary parameters of cortical bone**	
Ct.Po^a^ (%)	Cortical porosity

^a^Ct.Po is calculated as follows: Vo.Ar/Ct.BV × 100

### Statistical analysis

Each value was shown as the mean value ± standard error. An F-test was conducted to analyze the differences between the normal and control groups using all tested parameters and between the control and the investigational drug administered groups using bone histomorphometric parameters. A Student’s t-test (for equal variances) or Aspin-Welch’s t-test (for unequal variances) were also conducted. Bartlett’s method was employed to test for the homogeneity of variance between the control and the investigational drug administered groups using serum biochemical parameters. In addition, multiple comparison tests employing Dunnett’s method (for equal variances) or Steel’s method (for unequal variances) were conducted. Significant differences were identified at *P* < 0.05 on a 2-tailed basis.

## Results

### Body weight and drug intake

The mean body weight at day 28, the end of disease model period, was 467 ± 6 g in the normal group and 278 ± 7 g in the control group. Thus, decreased body weight was observed during the disease model period. However, there was no difference between the control group and each of the treatment groups during the treatment period. The mean body weight at day 56 was 340 ± 17 g in the control group, 324 ± 16, 343 ± 16, and 332 ± 10 g in the 0.5%, 1.5%, and 5% PA21 groups, 354 ± 12 and 335 ± 11 g in the 0.6% and 2% sevelamer groups, and 348 ± 15 and 345 ± 10 g in the 0.6% and 2% lanthanum groups, respectively. During the treatment period, the mean intakes of the investigated drugs per day were 240, 814, and 2821 mg/kg/day in the 0.5%, 1.5%, and 5% PA21 groups, 318 and 1059 mg/kg/day in the 0.6% and 2% sevelamer hydrochloride groups, and 308 and 1088 mg/kg/day in the 0.6% and 2% lanthanum carbonate hydrate groups, respectively.

### Clinical signs

In the treatment period, wasting, decreases in feces, piloerection, bradypnea, solid fur (nose), subnormal temperature, and abdominal distention were observed; however, the changes were due to progressive renal failure and not related to the investigated drugs.

### Blood biochemistry

**Fig 1:**
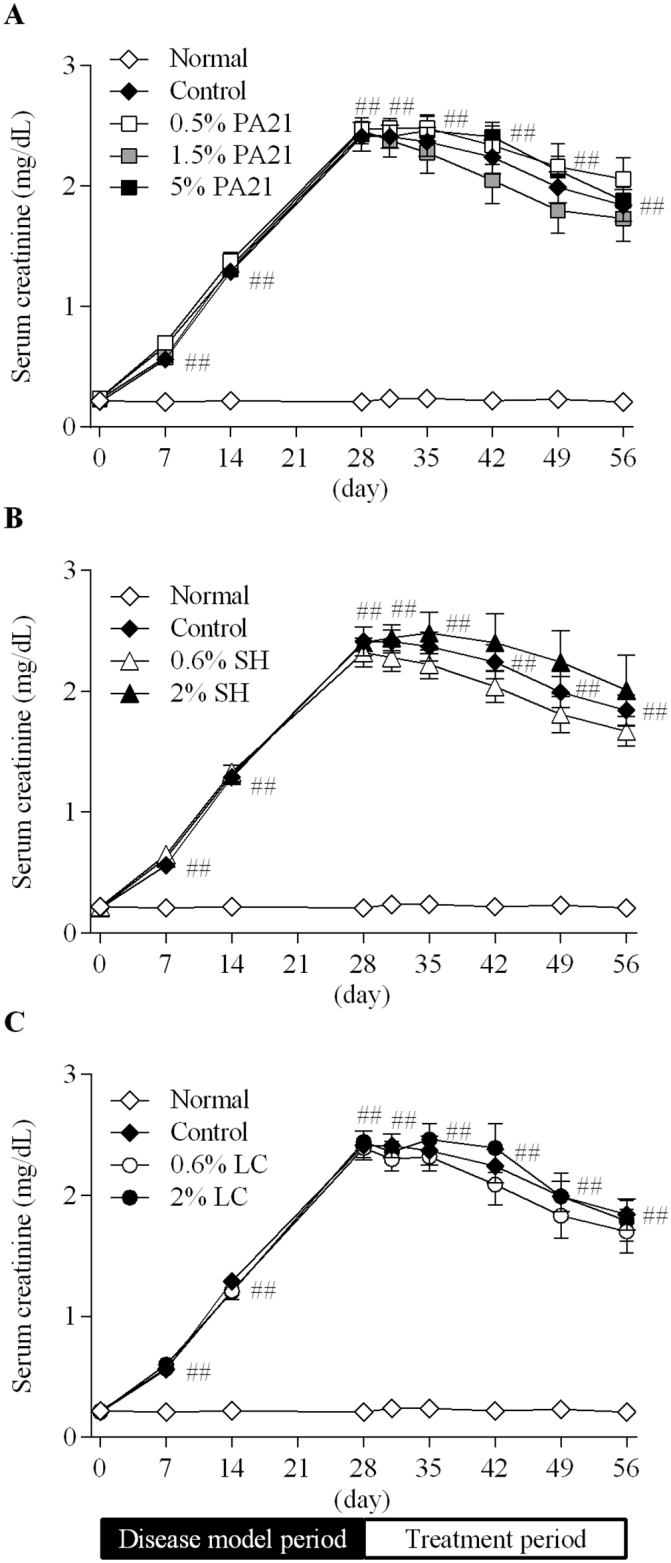
The effects of (A) PA21, (B) sevelamer hydrochloride, and (C) lanthanum carbonate hydrate on serum creatinine level. At day 29, administration of the investigated drugs was started. Each dot in the figures shows the mean value ± standard error of eight to 10 animals. SH, sevelamer hydrochloride; LC, lanthanum carbonate hydrate. ##*P* < 0.01, Aspin-Welch’s t-test between the normal and control groups.

Serum creatinine levels in the control group significantly increased during the disease model period (day 7 to day 28), and then maintained a significantly high level until the end of the study (Fig 1). SUN levels showed mostly the same changes as those of serum creatinine levels (S1 Fig). During the treatment period, there were no differences in the AUC_0–28 day_ of these parameters between the control group and each of the treatment groups (Table 3).

**Table 3:** AUC_0–28 day_ of serum biochemical parameters during treatment period.

Group	N	AUC_0–28 day_				
		Creatinine (mg·day/dL)	SUN (mg·day/dL)	Phosphorus (mg·day/dL)	Calcium (mg·day/dL)	PTH (× 10^4^ pg·day/mL)
Normal	10	6.34 ± 0.15	523 ± 12	210.9 ± 3.0	294.4 ± 1.9	0.233 ± 0.048
Control	10	61.09 ± 3.33^a^	4007 ± 292^a^	295.7 ± 14.6^a^	283.6 ± 4.7	5.225 ± 1.036^a^
0.5% PA21	9	64.75 ± 3.58	4240 ± 332	281.5 ± 14.5	286.9 ± 6.8	5.568 ± 0.944
1.5% PA21	10	57.52 ± 4.69	3735 ± 380	229.7 ± 9.4^b^	299.6 ± 4.0	3.346 ± 0.807
5% PA21	10	63.96 ± 2.76	3707 ± 163	160.5 ± 3.9^c^	298.8 ± 4.3	1.502 ± 0.380^c^
0.6% SH	8	56.41 ± 3.57	3857 ± 281	235.7 ± 10.3^c^	297.8 ± 4.0	2.433 ± 0.927
2% SH	10	65.26 ± 5.60	4757 ± 420	189.3 ± 7.4^c^	310.8 ± 5.7^c^	0.666 ± 0.182^c^
0.6% LC	10	57.76 ± 4.26	3862 ± 326	256.1 ± 9.1^b^	293.8 ± 5.8	3.987 ± 0.917
2% LC	10	62.37 ± 4.25	4161 ± 271	196.1 ± 7.3^c^	308.8 ± 4.1^c^	1.277 ± 0.185^c^

Each value was shown as the mean value ± standard error. SH, sevelamer hydrochloride; LC, lanthanum carbonate hydrate.

^*a*^*P* < 0.01, Aspin-Welch’s t-test between the normal and control groups.

^*b*^*P* < 0.05 and

^*c*^*P* < 0.01, Dunnett’s or Steel’s multiple comparison tests between the control and the investigated drug administered groups.

**Fig 2:**
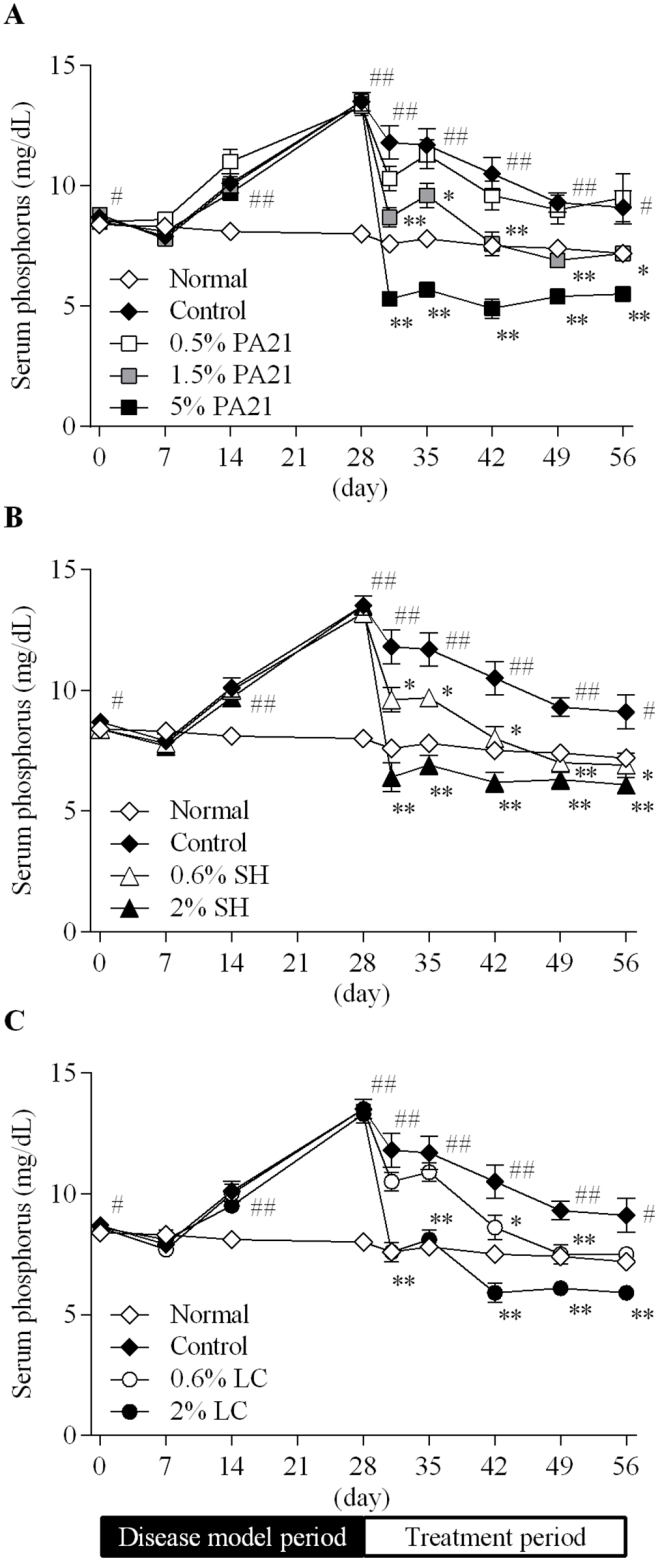
The effects of (A) PA21, (B) sevelamer hydrochloride, and (C) lanthanum carbonate hydrate on serum phosphorus level. At day 29, administration of the investigated drugs was started. Each dot in the figures shows the mean value ± standard error of eight to 10 animals. SH, sevelamer hydrochloride; LC, lanthanum carbonate hydrate. #*P* < 0.05 and ##*P* < 0.01, Student’s or Aspin-Welch’s t-test between the normal and control groups. *P < 0.05 and **P < 0.01, Dunnett’s or Steel’s multiple comparison tests between the control and the investigated drug administered groups.

Serum phosphorus level in the control group significantly increased during the disease model period (Fig 2). It remained significantly high. PA21, sevelamer hydrochloride, and lanthanum carbonate hydrate treatment dose-dependently decreased the serum phosphorus level. The AUC_0–28 day_ of the serum phosphorus level during the treatment period was dose-dependently low (Table 3).

**Fig 3:**
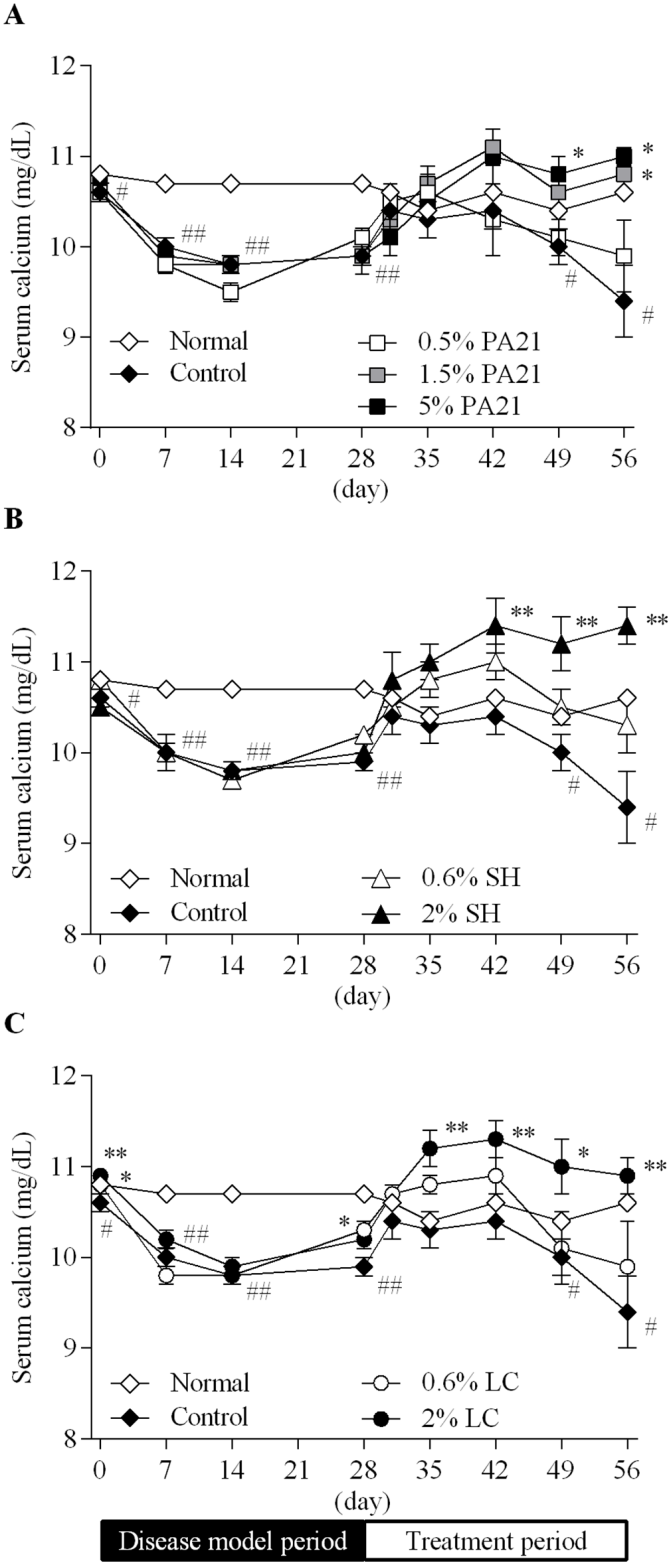
The effects of (A) PA21, (B) sevelamer hydrochloride, and (C) lanthanum carbonate hydrate on serum calcium level. At day 29, administration of the investigated drugs was started. Each dot in the figures shows the mean value ± standard error of eight to 10 animals. SH, sevelamer hydrochloride; LC, lanthanum carbonate hydrate. #*P* < 0.05 and ##*P* < 0.01, Student’s or Aspin-Welch’s t-test between the normal and control groups. *P < 0.05 and **P < 0.01, Dunnett’s or Steel’s multiple comparison tests between the control and the investigated drug administered groups.

Serum calcium level of the control group significantly decreased during the disease model period (Fig 3). In the high-dose treatment groups of PA21, sevelamer hydrochloride, and lanthanum carbonate hydrate, serum calcium level increased above the normal level. No significant increase of the AUC_0–28 day_ for serum calcium level in the 5% PA21 group was observed while the AUC_0–28 day_ in the 2% sevelamer hydrochloride and lanthanum carbonate hydrate groups increased significantly (Table 3).

**Fig 4:**
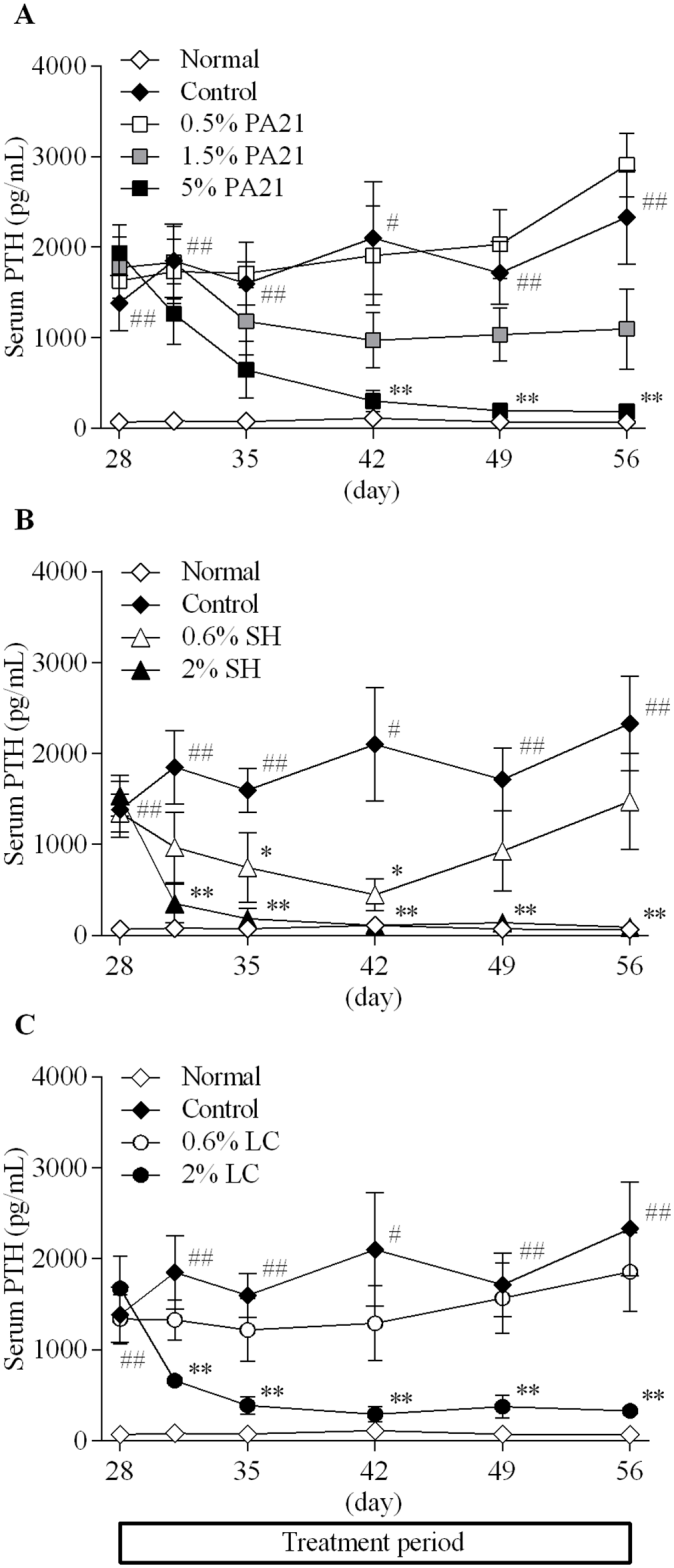
The effects of (A) PA21, (B) sevelamer hydrochloride, and (C) lanthanum carbonate hydrate on serum PTH level. Each dot in the figures shows the mean value ± standard error of eight to 10 animals. SH, sevelamer hydrochloride; LC, lanthanum carbonate hydrate. #*P* < 0.05 and ##*P* < 0.01, Aspin-Welch’s t-test between the normal and control groups. *P < 0.05 and **P < 0.01, Dunnett’s or Steel’s multiple comparison tests between the control and the investigated drug administered groups.

Serum PTH level in the control group was maintained at a significantly high level throughout the study (Fig 4). Serum PTH level in the treatment period showed a dose-dependent decrease in the PA21, sevelamer hydrochloride and lanthanum carbonate hydrate groups and the AUC_0–28 day_ of these parameters declined dose-dependently (Table 3).

### Bone histomorphometry

**Fig 5:**
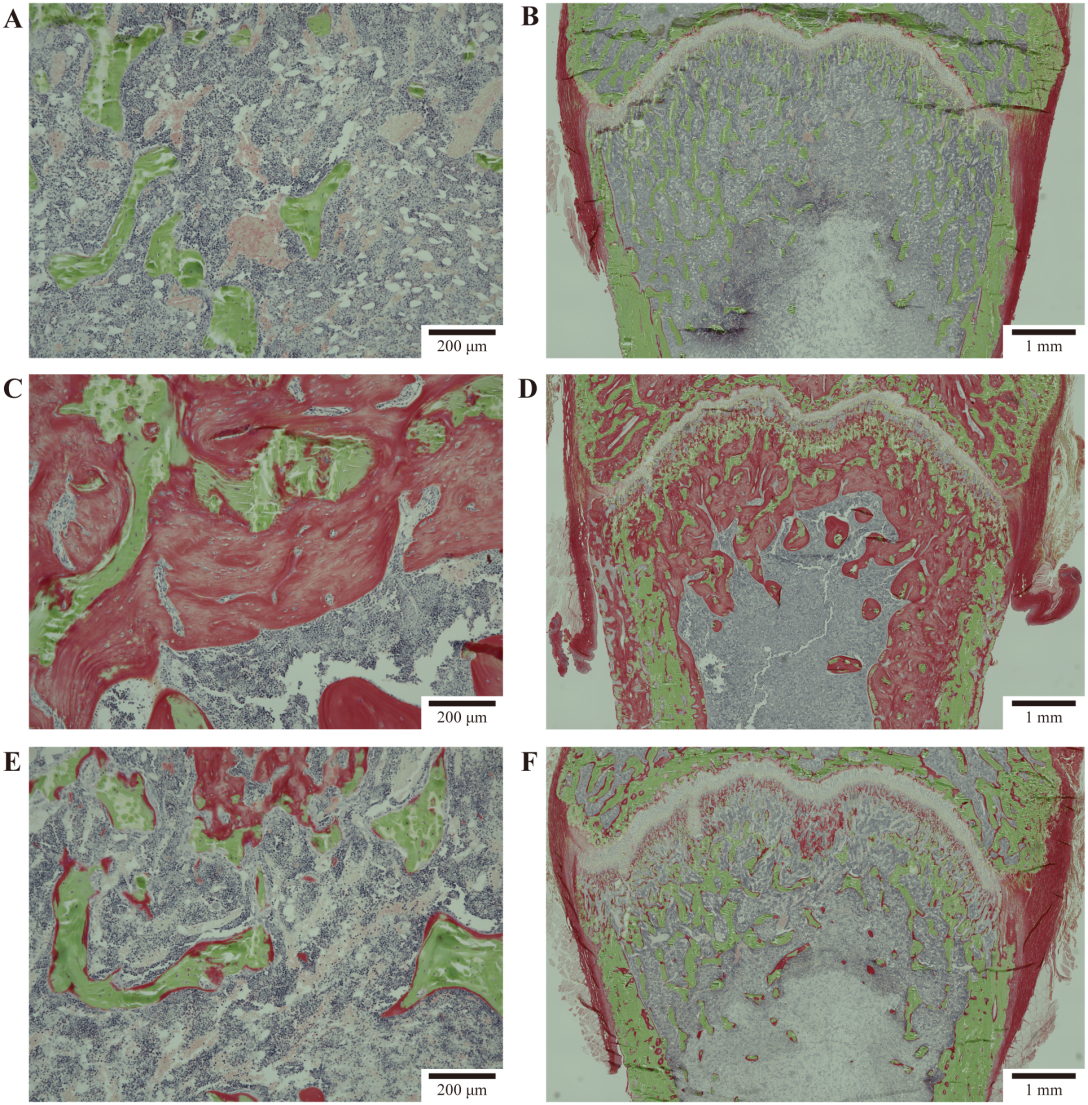
Representative images of the proximal femur of normal or CRF rats after four weeks of treatment with normal or 5% PA21 containing diet. The sections (6 μm) were cut and stained with a Villanueva-Goldner stain for bright-field microscopy. Representative images (low and high magnification) from some groups are presented. (A) Normal group (low magnification), (B) normal group (high magnification), (C) control group (low magnification), (D) control group (high magnification), (E) 5% PA21 group (low magnification), and (F) 5% PA21 group (high magnification). These sections demonstrate mature bone (yellowish green), immature (red) and other bone structure.

**Fig 6:**
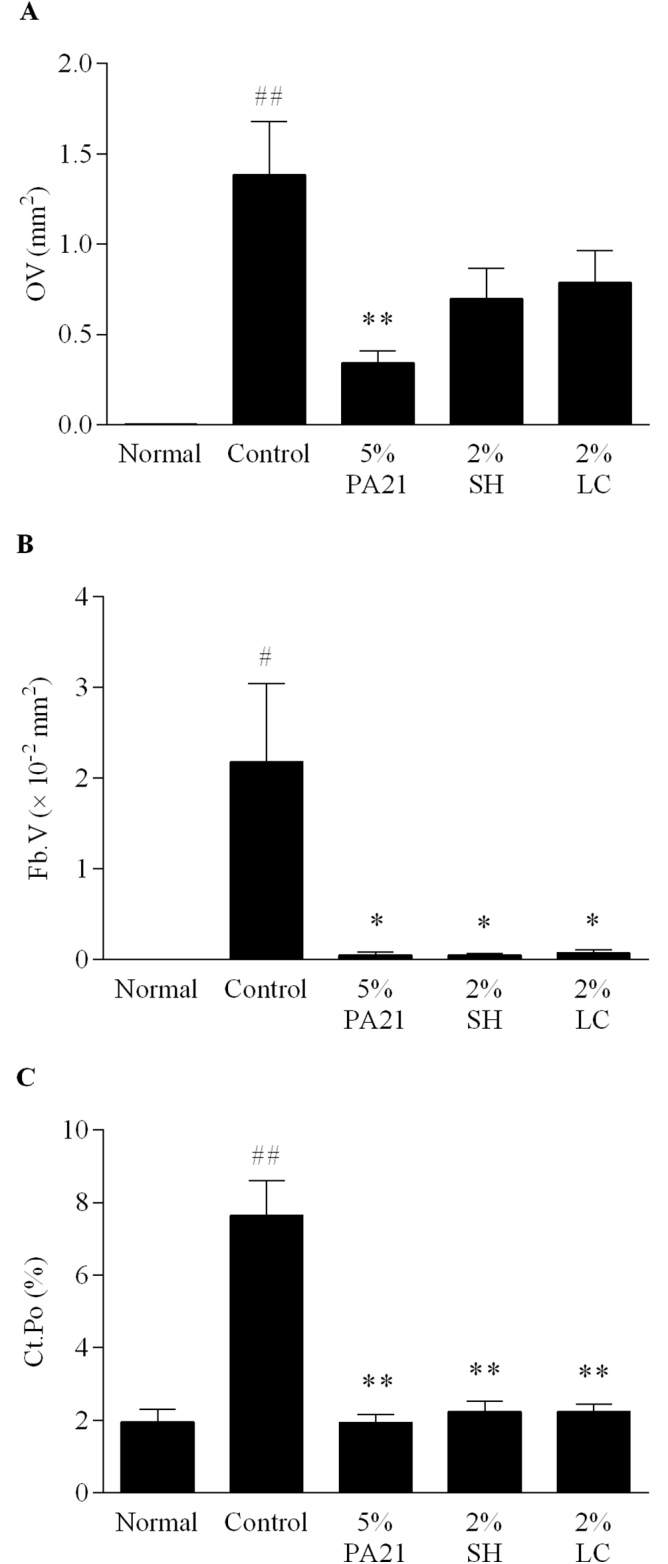
Effects of PA21, sevelamer hydrochloride, and lanthanum carbonate hydrate on (A) OV, (B) Fb.V, and (C) Ct.Po. Each column of the figures shows the mean value ± standard error of nine to 10 animals. SH, sevelamer hydrochloride; LC, lanthanum carbonate hydrate. #*P* < 0.05 and ##*P* < 0.01, Aspin-Welch’s t-test between the normal and control groups. *P < 0.05 and **P < 0.01, Aspin-Welch’s t-test between the control and the investigated drug administered groups.

Representative histopathological images of the distal femur of rats in the normal and the control groups, as well as in the 5% PA21 group are shown in Fig 5, and the results of bone histomorphometry are shown in Fig 6 and Tables 4 and 5.

**Table 4:** Effects of PA21, sevelamer hydrochloride, and lanthanum carbonate hydrate on trabecular bone histomorphometric parameters.

Group	Bone hisomorphometric parameters								
	BS (mm)	BV (mm^2^)	OV (mm^2^)	Ob.S (mm)	Oc.S (mm)	Fb.V (× 10^−2^ mm^2^)	OV/BV (%)	Ob.S/BS (%)	Oc.S/BS (%)
Normal	8.90 ± 1.03	0.224 ± 0.038	0.004 ± 0.001	0.594 ± 0.131	0.2492 ± 0.0379	0.000 ± 0.000	1.90 ± 0.40	6.56 ± 1.55	2.848 ± 0.353
Control	22.69 ± 3.78^b^	1.754 ± 0.316^b^	1.386 ± 0.293^b^	3.022 ± 0.621^b^	0.2865 ± 0.1510	2.180 ± 0.864^a^	73.57 ± 8.44^b^	14.58 ± 2.40^a^	1.040 ± 0.404^b^
5% PA21	14.63 ± 2.05	0.705 ± 0.136^d^	0.342 ± 0.067^d^	0.816 ± 0.238^d^	0.3567 ± 0.0880	0.052 ± 0.031^c^	53.82 ± 7.59	5.87 ± 1.88^c^	2.180 ± 0.386
2% SH	18.22 ± 2.06	1.053 ± 0.204	0.697 ± 0.171	1.071 ± 0.284^c^	0.3836 ± 0.1496	0.048 ± 0.018^c^	63.61 ± 8.21	5.70 ± 1.19^d^	2.006 ± 0.690
2% LC	16.95 ± 1.95	1.214 ± 0.225	0.788 ± 0.178	1.328 ± 0.313^c^	0.3564 ± 0.1337	0.076 ± 0.031^c^	58.22 ± 7.84	10.06 ± 3.09	2.115 ± 0.730

Each value was shown as the mean value ± standard error of ten animals. SH, sevelamer hydrochloride; LC, lanthanum carbonate hydrate.

^*a*^*P* < 0.05 and

^*b*^*P* < 0.01, Student’s or Aspin-Welch’s t-test between the normal and control groups.

^*c*^*P* < 0.05 and

^*d*^*P* < 0.01, Student’s or Aspin-Welch’s t-test between the control and investigated drug administered groups.

**Table 5:** Effects of PA21, sevelamer hydrochloride, and lanthanum carbonate hydrate on cortical bone histomorphometric parameters.

Group	Bone hisomorphometric parameters		
	Ct.BV (mm^2^)	Vo.Ar (mm^2^)	Ct.Po (%)
Normal	1.653 ± 0.031	0.0324 ± 0.0058	1.951 ± 0.343
Control	3.254 ± 0.382^a^	0.2521 ± 0.0428^a^	7.644 ± 0.964^a^
5% PA21	2.206 ± 0.107^b^	0.0424 ± 0.0050^c^	1.939 ± 0.222^c^
2% SH	2.296 ± 0.133^b^	0.0510 ± 0.0068^c^	2.236 ± 0.295^c^
2% LC	2.283 ± 0.077^b^	0.0517 ± 0.0063^c^	2.235 ± 0.219^c^

Each value was shown as the mean value ± standard error of ten animals. SH, sevelamer hydrochloride; LC, lanthanum carbonate hydrate.

^*a*^*P* < 0.01, Aspin-Welch’s t-test between the normal and control groups.

^*b*^*P* < 0.05 and

^*c*^*P* < 0.01, Aspin-Welch’s t-test between the control and investigated drug administered groups.

In the control group, BS, BV, OV, Ob.S, Fb.V, OV/BV, Ob.S/BS, Ct.BV, Vo.Ar, and Ct.Po showed a significant increase, and Oc.S/BS showed a significant decrease (Fig 6 and Tables 4 and 5). In the 5% PA21, 2% sevelamer hydrochloride, and 2% lanthanum carbonate hydrate groups, all parameters except for Oc.S showed improvement. A significant improvement was found for BV, OV, Ob.S, Fb.V, Ob.S/BS, Ct.BV, Vo.Ar, and Ct.Po in the 5% PA21 group. Ob.S, Fb.V, Ob.S/BS, Ct.BV, Vo.Ar, and Ct.Po in the 2% sevelamer hydrochloride group and Ob.S, Fb.V, Ct.BV, Vo.Ar, and Ct.Po in the 2% lanthanum carbonate hydrate group showed a significant improvement.

## Discussion

PA21, a new phosphate binder, suppressed osteoid formation, fibrosis, and porousness; decreased serum phosphorus and PTH levels; and significantly suppressed OV and Fb.V of trabecular bones and Ct.Po of cortical bones. Thus, PA21 inhibited the progression of ROD by decreasing serum phosphorus levels.

Various CRF animal models are used to evaluate improving trends in symptoms of CKD-MBD [17, 18, 19]. The adenine-induced CRF rat model is a popular model of CKD-MBD and shows SHPT, ectopic calcification, ROD, and hyperphosphatemia. Thus, it is used in the pharmacometric studies of phosphate binders [20, 21]. PA21 improved hyperphosphatemia and vascular calcification in this model [15, 16]. However, its effect on ROD in this model is not reported. It is difficult to define ROD as specific lesions, as bone lesions in CKD-MBD are transferred in various ways under the influence of many factors. However, ROD was determined to have developed because serum concentrations of PTH, which modulates bone turnover, increased and many bone histomorphometric parameters fluctuated in this study. Especially, the changes in OV, Fb.V, and Ct.Po, which is evaluated in the pharmacometric analyses of other drugs [21, 22], were observed in this study and are similar to those studies. Therefore, pharmacometric analysis with this model is thought to be suitable for assessing ROD in CKD-MBD.

In the control group, Ob.S/BS increased significantly; hence, it is suggested that bone formation was promoted and osteoblasts were hyperactive. Oc.S/BS decreased significantly because Oc.S remained unchanged while BS was elevated by increasing BV, and bone resorption relatively decreased. In contrast, fibrosis tissue, an indicator of high bone turnover, was observed, suggesting that bone resorption and formation were promoted. This could be a reason for the contrary results obtained, in that bone histomorphometry was performed at a time when acceleration of bone resorption changed to suppression; however, the details are unknown. Notwithstanding, it was suggested that bone cells were activated and bone resorption and formation were accelerated because osteitis fibrosa was observed. Additionally, the significant increase in OV/BV and the observed lesion of osteomalacia indicated abnormal bone calcification. Increased Ct.BV and Ct.Po in the cortical bone supported the development of these abnormalities. Thus, this was the model in which bone cell hyperactivity induced by PTH hypersecretion and delayed bone calcification were observed.

The 5% PA21 treatment significantly reduced Fb.V to the normal level, indicating that PA21 ameliorated the lesion of osteitis fibrosa strongly. Furthermore, the decreased Ob.S/BS and increased Oc.S/BS observed suggests that bone cell activity was normalized, and the balance between bone resorption and formation improved, corresponding with the tendency for improvement of Ct.BV and the significant amelioration of Ct.Po in the cortical bone.

Contrary to expectations, however, OV/BV was not improved and PA21 did not suppress the delayed bone calcification inferred from the increased OV/BV. OV/BV did not change mathematically because PA21 treatment improved the OV to the same level as BV. Nevertheless, because these two parameters were close to normal levels, there is no doubt that PA21 improved abnormal bone structure.

In this study, the sequential change in bone metabolism is unclear because bone pathology was evaluated at the end of treatment period; thus, temporal measurements of bone metabolism markers, such as alkaline phosphatase and tartrate-resistant acid phosphatase 5b, which are the markers of bone formation and resorption, respectively [23], is interesting since it may clarify the detailed changes in bone metabolism and help to elucidate the reason why the contrary results were observed regarding bone resorption.

FGF23 is associated with bone metabolism, and inhibits bone mineralization though it is uncertain whether it acts directly or indirectly [24]. Phan et al. reported that treatments with 5% PA21 and 2% lanthanum carbonate decreased serum FGF23 in CRF rats, but a significant decrease was observed only with 5% PA21 treatment compared with that in CRF control [16, 25]. Therefore, there may be a difference in the decreased serum FGF23 between the PA21 and lanthanum carbonate treatment in this study, which resulted in significant suppression of OV with 5% PA21 treatment. However, the FGF23 level was not measured in this study, and the details are therefore unclear. Measurement of serum FGF23 is considered important to confirm this relationship.

Speedy recovery of serum calcium levels was observed after the disease model period in this study. However, the change in serum calcium was not consistent with the change in serum PTH, which is a calcium controlling factor, and it was difficult to explain the underlying reason. Even though serum creatinine and SUN gradually decreased in the treatment period as a whole, this reduction was not enough to improve renal function for normalizing the serum calcium level. On the other hand, 1,25(OH)_2_D_3_, which controls serum calcium levels by promoting calcium absorption from the intestines and increasing bone resorption [26], and bone metabolism markers, which help us estimate the inflow and outflow of calcium in bone through bone metabolism, were not measured. Therefore, the reason remains unknown. We want to examine serum 1,25(OH)_2_D_3_ and bone metabolism markers because this question may be solved by sequential measurement of these parameters.

In this study, 5% PA21 treatment decreased serum phosphorus and PTH levels and ameliorated bone histomorphometric parameters. The same improvements were observed in the 2% sevelamer hydrochloride and the lanthanum carbonate hydrate treatments. These results indicated that PA21 had a similar effect to that of the existing drugs. The AUC_0–28 day_ of serum calcium levels in the 2% sevelamer hydrochloride and the lanthanum carbonate hydrate group was significantly increased. While the detailed reason why these non-calcium-based drugs increased the serum calcium level is uncertain, the adverse effect of hypercalcemia has been reported in these drugs [2]; this was also observed in this study.

Phosphate binders inhibit phosphate absorption into the body by binding dietary phosphates in the GI tract and excreting them through the feces. From the point of view of this mechanism, the dosage of phosphate binders should not be calculated as the amount of phosphate binders per body weight but as the ratio of the quantity of phosphate binders to phosphorus intake. In this study, food consumption and PA21 intake in the 5% PA21 group, in which several parameters were improved significantly, was 56.4 g/kg/day and 2821 mg/kg/day, respectively. The phosphorus content of used food was 1.05% and the ratio of the quantity of PA21 to phosphorus intake in these rats was computed at 5.0. Phosphorus intake per day is said to be about 1200 mg/day in healthy adults and 800–1000 mg/day in dialysis patients, whose phosphorus intake is restricted [27, 28]. The ratios of PA21 to phosphorus intake are 4.2 (healthy adults) and 5.0–6.3 (dialysis patients) when calculated with a dose of 5.0 g/day, at which serum phosphorus level was significantly decreased in clinical practice [14], which are similar to that observed in this study. Thus, the dose of PA21 used in this study is similar to that used in clinical practice and is thought to be the appropriate dose.

Phosphate binders used in Japan showed a sufficient reducing effect on serum phosphorus levels in clinical practice. On the other hand, the adverse effects associated with the character of each drug are reported. Calcium carbonate is inexpensive, but can cause ectopic calcification, which is common with calcium-based drugs [29]. Serum calcium levels should be managed carefully because calcification of the coronary artery is strongly associated with mortality [5, 6]. Sevelamer hydrochloride is a non-calcium-based polymer that acts as a phosphate binder. It requires a high pill burden, is insoluble, and causes adverse effects such as abdominal distension and constipation [30, 31]. Lanthanum carbonate hydrate is a non-calcium-based phosphate binder, and it causes many GI adverse effects such as nausea and vomiting [32, 33]. Lanthanum does not exist in the human body; thus, its accumulation in the body is of great concern. While long-term treatment with lanthanum carbonate hydrate leads to an accumulation in the bone [34, 35], there is no report about the abnormality of bone formation and histology as a result of this accumulation. Longer-term safety data on lanthanum accumulation is yet to be produced. PA21 is a non-calcium-based phosphate binder. Thus, it does not cause ectopic calcification; rather, it inhibits vascular calcification in CRF rats [15, 16].

From the results of a clinical study, diarrhea was observed as a side effect of the PA21; however, it was generally mild and transient [36]. Furthermore, PA21 contains only iron, which is an essential element in the body, as a metal ingredient and it is thought that there is little concern about long-term accumulation.

In conclusion, PA21 ameliorated various symptoms such as hyperphosphatemia and SHPT. Additionally, it improved bone formation and resorption by suppressing bone cell hyperactivity, resulting in a reduction of bone tissue lesions. Although further research on bone metabolism is necessary to elucidate a more detailed mechanism for this improvement, PA21 can be expected to become a new phosphate binder of choice as it has positive effects similar to those of existing phosphate binders.

## Supporting information

S1 Fig The effects of (A) PA21, (B) sevelamer hydrochloride, and (C) lanthanum carbonate hydrate on SUN level. At day 29, administration of the investigated drugs was started. Each dot in the figures shows the mean value ± standard error of eight to 10 animals. SH, sevelamer hydrochloride; LC, lanthanum carbonate hydrate. ##*P* < 0.01, Aspin-Welch’s t-test between the normal and control groups.
(TIF)
