# Supplementary figures and images for: PA21, a novel phosphate binder, improves renal osteodystrophy in rats with chronic renal failure
Source: PLoS One. 2017 Jul 13;12(7):e0180430. doi: 10.1371/journal.pone.0180430 (PMC5509238; doi:10.1371/journal.pone.0180430)

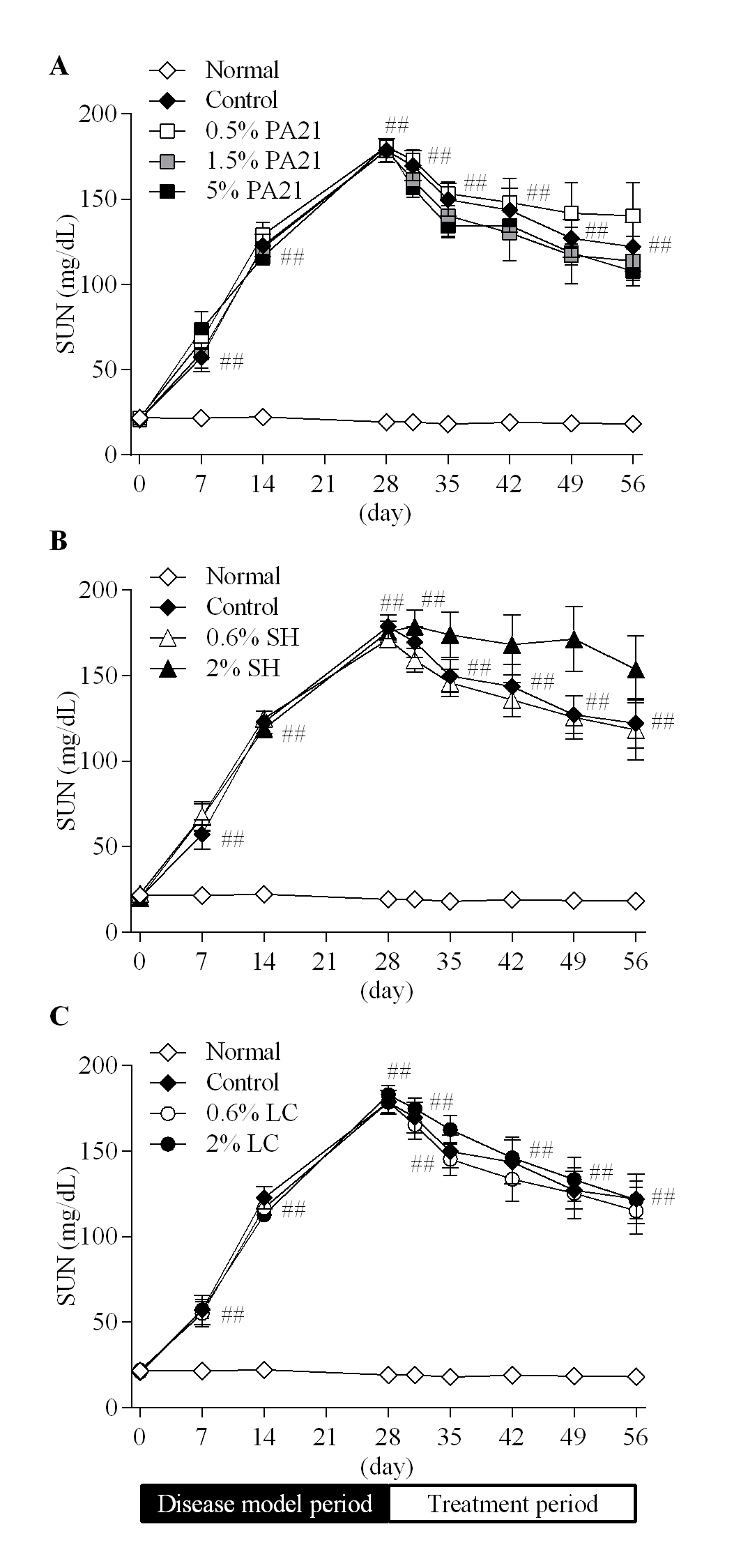

Supplement: S1 Fig — At day 29, administration of the investigated drugs was started. Each dot in the figures shows the mean value ± standard error of eight to 10 animals. SH, sevelamer hydrochloride; LC, lanthanum carbonate hydrate. ##P < 0.01, Aspin-Welch’s t-test between the normal and control groups. (TIF) [file pone.0180430.s001.tif]
